# The quality of sleep in systemic sclerosis patients is determined not only by the underlying disease but also by many internal and external factors

**DOI:** 10.1590/1806-9282.20240946

**Published:** 2024-09-30

**Authors:** Josef Finsterer

**Affiliations:** 1 Neurology & Neurophysiology Center, Department of Neurology – Vienna, Austria.

Dear Editor,

We read with interest the article by Santos et al. on a cross-sectional study of sleep quality, assessed with the Pittsburgh Sleep Quality Index (PSQI), and its impact on quality of life, assessed with the 12-item Short-Form Health Survey (SFHS), and disability, assessed with the Scleroderma Health Assessment Questionnaire (SHAQ), in 50 patients with systemic sclerosis (SS)^1^. It was found that 84% of SS patients had poor sleep quality and 20% had excessive daytime sleepiness^1^. There was a negative correlation between the PSQI and the physical and psychological components of the SFHS and a positive correlation with the SHAQ^1^. It was concluded that poor sleep quality is common in SS patients and has a negative impact on quality of life and level of disability^1^. The study is impressive, but two points need to be discussed.

The first point is that sleep quality in patients with SS depends not only on the disease stage, degree of multi-organ involvement, and functional disability of SS but also on many additional intrinsic and extrinsic factors that influence sleep quality in general and should be included in the analysis.

Intrinsic factors that may influence sleep quality include the patient’s genetic background, the individual’s character, and temperament, the individual’s psychological response to SS, the processing of daytime experiences before and during sleep, the patient’s comorbidities, whether the time a patient goes to bed is fixed or variable, and the acute and chronic stress levels the patient is undergoing. In terms of psychological response, the quality of sleep may depend on the person’s attitude toward his illness and the coping strategies he develops to deal with it. A person’s character and psychological constitution can greatly influence the amount of stress a patient produces for themselves. Patients who react with anxiety and depression may develop insomnia. Comorbidities that can affect sleep quality include nocturnal pain, the presence of sleep apnea syndrome, restless legs, sweating, fever, cough, the degree of sympathetic/parasympathetic nervous system activation, the presence of nocturia, and the presence of nocturnal seizures.

External factors that determine sleep quality include the environment in which an SS patient sleeps, climate zone, social status and environment, comedication, type of food and drink (e.g., coffee, Red Bull, cola, and other stimulant or illicit substances, and heavy and late meals can disrupt sleep), and timing of food and water intake. We should know how many patients were alcohol dependent, regular users of tetrahydrocannabinol, or smokers. As for the environment in which the patient sleeps, the quality of sleep can be strongly influenced by the noise level (e.g., refrigerator, air conditioning, cars on the street, noisy neighbors, screaming children, barking dogs, and ringing phones) in the bedroom at the beginning and during sleep; the brightness of the room; exposure to the screens of a cell phone, TV, laptop, or tablet; the level of electrosmog, the temperature, draft and humidity in the bedroom; and the air quality in the bedroom (level of air pollution in an urban or rural area). As far as nighttime concomitant medications are concerned, sleep can be highly dependent on the type and amount of sedative or activating medication taken before bedtime.

The second point is that sleep quality was assessed by a subjective assessment and not by an objective examination. The disadvantages of subjective assessment of sleep quality may differ significantly compared to objective measures, and the subjective assessment may depend heavily on cognitive abilities and memory functions that were not assessed before the PSQI was administered. The only objective assessment of sleep quality would be polysomnography, which can more accurately answer all the questions asked in the PSQI.

In conclusion, it can be said that this interesting study has limitations that relativize the results and their interpretation. Addressing these limitations could strengthen the conclusions and corroborate the study’s message. Before attributing sleep disorders in SS patients solely to the underlying disease, the entire spectrum of influences on sleep quality should be considered.
